# Anévrysme congénital géant intra péricardique de l’auricule gauche: à propos d’un cas avec revue de la littérature

**DOI:** 10.11604/pamj.2016.24.225.10012

**Published:** 2016-07-12

**Authors:** Bouchra Zhari, Habib Bellamlih, Hassan Boumdine, Touriya Amil, Mehdi Bamous, Hassan En-nouali

**Affiliations:** 1 Service d’Imagerie Médicale, Hôpital Militaire d’Instruction Mohamed V, Université Mohamed V Rabat, Maroc; 2 Service de Chirurgie cardiovasculaire, Hôpital Militaire d’Instruction Mohamed V, Université Mohamed V Rabat, Maroc

**Keywords:** Anévrysme congénital auriculaire gauche, coroscanner, malformation cardiaque, Left atrial appendage aneurysm, coronary CT angiography, cardiac malformation

## Abstract

L'anévrysme auriculaire gauche est une anomalie cardiaque très rare. Il peut être d'origine congénitale ou acquise; secondaire à des processus inflammatoires ou dégénératifs. La plupart des cas sont asymptomatiques. La prévalence de ces lésions chez des patients d'âge pédiatrique a été très rarement décrite. Comme il peut provoquer des arythmies potentiellement mortelles ou des thrombus, le traitement chirurgical est nécessaire immédiatement après le diagnostic. Dans cet article, nous présentons le cas d'un garçon de 14 ans qui rapporte une dyspnée rapidement progressive, des palpitations, avec une notion de vertiges répétitifs et lipothymies, chez qui on découvre un anévrysme auriculaire gauche congénital. Le diagnostic a été basé sur les données du Coro scanner. Le patient a été traité avec succès par résection chirurgicale de l'anévrysme.

## Abstract (en)

Left atrial appendage aneurysm is a very rare heart anomaly. It may be congenital or acquired, secondary to inflammatory or degenerative processes. Most cases are asymptomatic. The prevalence of these lesions in pediatric age has been very rarely reported. As it can cause potentially fatal arrhythmias or thrombus, surgery is required immediately after diagnosis. This study reports the case of a 14-year-old boy with rapidly progressive dyspnea, palpitations, sensation of repetitive dizziness and fainting, in whom congenital left atrial appendage aneurysm was detected. Diagnosis was based on coronary CTA data. The patient was successfully treated with surgical resection of the aneurysm.

## Introduction

L’anévrysme auriculaire gauche (AAG) est une anomalie cardiaque extrêmement rare. Il peut se produire soit secondairement à une maladie de la valve mitrale ou un processus dégénératif de la paroi atriale. Lorsqu’il est congénital, la plupart des cas sont asymptomatiques dans l'enfance [1]. Les patients peuvent présenter une variété de symptômes à l’âge adulte, les plus fréquents étant l'apparition d'une dyspnée, une arythmie et tachycardie auriculaire, rarement une douleur thoracique supra ventriculaire, ou des signes d’embolie systémique [2]. Différentes techniques d'imagerie sont utiles dans le diagnostic et permettent le diagnostic différentiel avec d'autres pathologies. Le traitement chirurgical est nécessaire immédiatement après le diagnostic, en raison des complications qui peuvent être dévastatrices, en particulier thromboembolique, dysfonctionnement cardiaque par compression de ses cavités et la mort soudaine.

## Patient et observation

**Figure 1:**
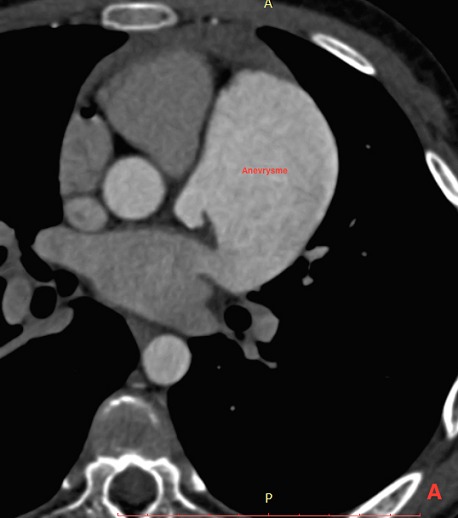
Coroscanner avec injection de PDC et Gatting cardiaque, en coupes axiale, montrant une ectasie de l’auricule gauche avec la présence d’une formation arrondie adjacente dont le rehaussement est synchrone de celle-ci, avec large pertuis de communication entre les deux: il s’agit d’un anévrysme géant développé à partir de l’auricule gauche, mesurant 70 x 50 mm de grands axes et refoulant le ventricule gauche; il présente un développement intra péricardique, sans defect du septum inter ventriculaire ou de thrombose intra cavitaire

**Figure 2:**
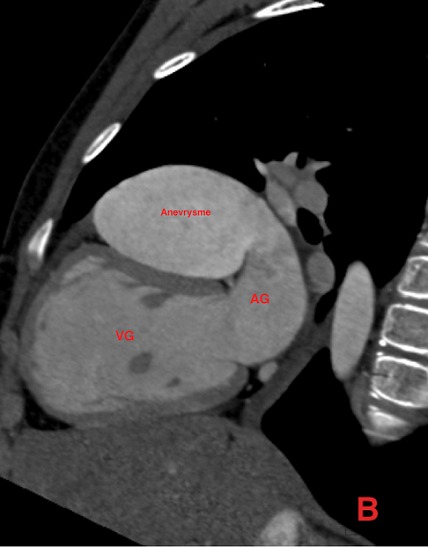
Coroscanner avec injection de PDC et Gatting cardiaque, en reconstruction sagittale, montrant la communication avec l’auricule gauche et l’effet sur le ventricule gauche

**Figure 3:**
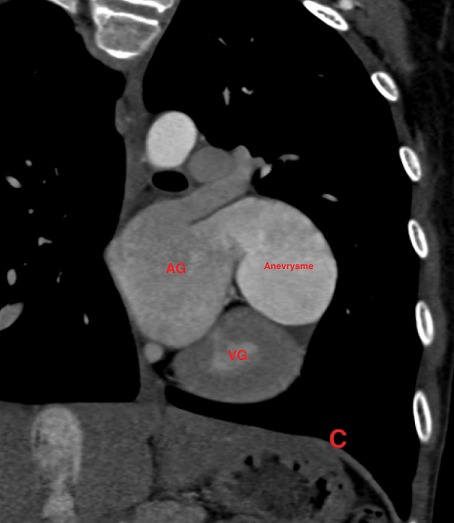
Coroscanner avec injection de PDC et Gattig cardiaque, en reconstruction coronale

**Figure 4:**
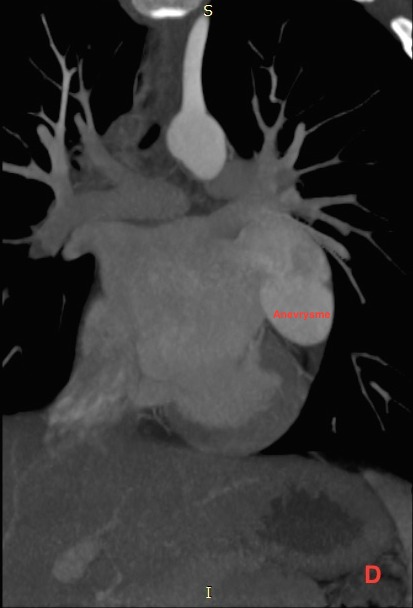
Coroscanner avec injection de PDC et Gatting cardiaque, en reconstruction MIP

**Figure 5:**
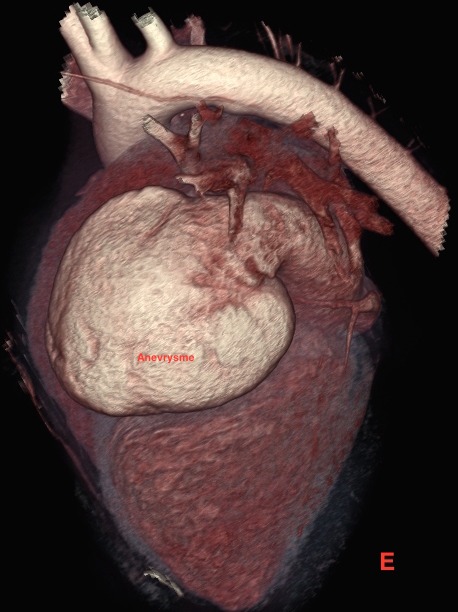
Coroscanner avec injection de PDC et Gatting cardiaque, en reconstruction VRT

**Figure 6:**
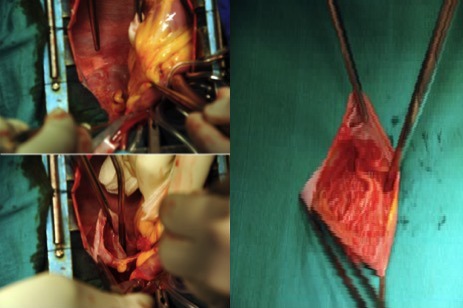
Champ chirurgical montrant l’anévrysme attaché à l’auricule gauche, avant et après exérèse

Il s’agit d’un patient de 14 ans sans antécédents pathologiques notables, qui rapporte une dyspnée rapidement progressive, des palpitations, avec une notion de vertiges répétitifs et lipothymies, le tout évoluant dans un cadre de faiblesse générale. L’examen cardiovasculaire était sans particularités. Le patient a bénéficié d’un ECG, qui a montré une tachycardie avec fibrillation auriculaire, et la radiographie thoracique avait objectivé un aspect proéminent du bord gauche du cœur avec cardiomégalie. Le complément par une échocardiographie transthoracique a révélé un aspect de pseudo cavité liquidienne reliée à l'oreillette gauche, accolée à la paroi antérolatérale du ventricule gauche, avec respect des autres structures cardiaque. Le doppler couleur et pulsé avait démontré la présence d’un flux sanguin à partir de l'oreillette gauche vers cette pseudo cavité, qui avait été retenue comme kyste péricardique. Le patient nous a été adressé pour la réalisation d’un coroscanner à visée diagnostique. La tomographie a montré un anévrysme géant de l’auricule gauche, de siège intra péricardique, mesuré dans son axe transversal à plus de 7 cm x 5 cm, sans défect du septum inter ventriculaire ou de thrombus intra cavitaire (Figure 1). Les reconstructions tridimensionnelles (Figure 2, Figure 3, Figure 4 et Figure 5) ont été réalisées afin d’illustrer les liaisons spatiales entre cet anévrysme, les artères coronaires et le ventricule gauche. Le patient a également bénéficié d’une angiographie des artères coronaires qui est revenue sans particularités coronariennes en dehors d’anomalies liées à la compression du cœur. Le diagnostic d’anévrysme congénital géant de l’auricule gauche a été retenu. En l’absence de traitement, les complications peuvent être dévastatrices, en particulier thromboembolique et la mort soudaine. Le jeune patient a été mis sous héparine de bas poids moléculaire à dose curative et fut adressé en consultation de chirurgie cardiovasculaire et thoracique, où une chirurgie d’exérèse a été réalisée (Figure 6) avec des suites opératoires satisfaisantes, et reprise du rythme sinus normal. Le patient s’est amélioré après le traitement, et la sortie fut réalisée le 10^ème^ jour du post opératoire.

## Discussion

L’anévrysme de l’auricule gauche (AG) est une anomalie extrêmement rare, avec moins de 100 cas rapportés dans la littérature [3]. Il est encore plus rare dans la population pédiatrique [4]. Il est défini comme une structure sacculaire (dilatation localisée ou diffuse), ayant pour origine la paroi auriculaire libre. La différence entre anévrysme et diverticule auriculaire est controversée, mais il semble qu’ils soient deux entités distinctes. Toutefois, il n’existe pas de consensus clair, de définition ou de distinction entre un anévrysme ou un diverticule de l’AG. Il peut être d'origine congénitale (faiblesse congénitale de la paroi auriculaire, résultant d'une dysplasie des bandes musculaires) ou acquise (élargissement de l’AG par maladie de la valve mitrale) [3]. De plus, l'absence de pathologie mitrale, un volume auriculaire normal et la présence de compression extrinsèque du VG suggéreraient l’origine congénitale [5]. Dans la littérature, la plupart des anévrysmes auriculaires congénitaux étaient extra péri cardiaques, liés à un défect du péricarde [6]. Chez notre patient, son siège était intra péricardique, et les résultats scannographiques et per opératoires ont démontré que le péricarde sus-jacente à l'anévrysme était intact. La majorité des patients sont asymptomatiques. Généralement, les AAG congénitaux se manifestent entre la 2^ème^ et la 4^ème^ décennie de la vie, mais ils peuvent être diagnostiqués de façon fortuite au cours d’une imagerie cardiaque de routine, à tout moment entre la vie fœtale et adulte [3, 4]. Lorsqu’ils se manifestent à l’âge adulte, les patients présentent une dyspnée à l’effort, palpitations, vertiges ou des phénomènes thromboemboliques; tandis que chez les nourrissons ou les enfants, l’AAG peut être la cause d’une détresse respiratoire, une tamponnade cardiaque ou d’une mort subite [6, 7]. Leur taille peut augmenter avec l’âge, avec parfois des anévrysmes géants au moment du diagnostic, ce qui augmente le risque de fibrillation auriculaire ainsi que de formation de thrombus. Parfois il peut être responsable de compression des cavités cardiaques, ayant pour conséquence un dysfonctionnement cardiaque. Actuellement, le diagnostic repose sur les progrès de l’imagerie basée sur différentes techniques d'imagerie non invasives telles que l’échocardiographie, l’échographie anténatale, le scanner ainsi que l’IRM cardiaques [8]. La radiographie thoracique montre souvent un élargissement de l’arc moyen gauche du cœur, faisant suspecter une anomalie auriculaire gauche [1].

L’échocardiographie trans-thoracique couplée au doppler couleur, est un examen de choix, non irradiant et inoffensif. Elle montre une masse liquidienne adjacente à la paroi auriculaire, et confirme la présence d’un flux sanguin entre l’AG et l’anévrysme, montre le pertuis et la présence d’éventuels thrombi. L’échocardiographie trans-oesophagienne est plus invasive et comporte un risque de rupture et d’embolie. La TDM et/ ou l’IRM cardiaque confirment le diagnostic en montrant l’anévrysme, précisent sa taille et celle de son collet ainsi que l’existence d’un éventuel thrombus. Ils permettent également d’étudier les rapports lésionnels avec les structures vasculaires adjacentes [7]. Le diagnostic différentiel se fait avec toute éventuelle structure liquidienne intra péricardique, adjacente aux cavités cardiaques gauches, telle que: le kyste péricardique, un anévrisme de l'artère coronaire, pseudo anévrisme du VG [5]. L'évolution naturelle de ces malformations reste incertaine. Ils sont souvent associés à des complications pouvant être dévastatrices, surtout thromboemboliques et l’arythmie cardiaque potentiellement mortelles. L'anévrisme peut également augmenter de taille avec le temps, ce qui augmente le risque de complications. Un anévrysme géant est défini par des mesures = 65 mm [1]. Bien que notre patient avait un tel anévrisme massif, il n'y avait pas d'épisodes thrombotiques ou emboliques. Par conséquent, le traitement chirurgical est nécessaire immédiatement après le diagnostic, même dans les cas asymptomatiques. Quand il est secondaire à une autre pathologie, le traitement de celle-ci devrait être envisagé L9; avant la cure de l’anévrysme. Cependant quand il est congénital, le seul traitement est la résection chirurgicale [9].

## Conclusion

L'anévrisme auriculaire gauche est une entité rare mais cliniquement importante. Le coroscanner a permis de faire aisément le diagnostic positif, tout en précisant ses rapports, pour un traitement approprié. L’échocardiographie trans-thoracique et l'IRM, restent cependant des techniques non-invasives de choix pour le diagnostic chez les patients présentant des symptômes de maladie cardiaque. Enfin, et compte tenu du fait de leur potentiel pour des complications plus dévastatrices, il est très important de savoir poser le diagnostic afin d’en faire l´excision chirurgicale au moment opportun.

## References

[1] Taori K, Deshmukh A, Sanyal R, Saini T, Sheorain V, Rathod J (2006). Giant Congenital intrapericardial left atrial aneurysm diagnosed by contrast-enhanced computed tomography. Acta Radiol.

[2] Munárriz A, Escribano E, Urchaga A, Olaz F, Beunza M, de La Fuente A, Cantabrana S, Sola T (2008). Congenital aneurysm of the left atrial appendage. Eur J Echocardiogr.

[3] Madan Raj Aryal (2014). Left Atrial appendage aneurysm: a systematic review of 82 cases. Echocardiography.

[4] Awasthy N, Tomar M, Radhakrishnan S, Shrivastava S, Iyer KS (2010). Symptomatic giant left atrial aneurysm in a child: a rare entity. Images Paediatr Cardiol.

[5] Fawn Atchison W, Kent Rehfeldt H (2011). Congenital Left Atrial Appendage Aneurysm. International anesthesia research society.

[6] Lu Wen-Yu, Huang Shu-Chien, Chen Shyh-Jye, Jhuang Jie-Yang, Chen Yung-Chuan, Wang Jou-Kou, Wu Mei-Hwan, Chen Chun-An (2012). Giant Congenital Left Atrial Aneurysm in an 11-Year-Old Boy.

[7] Abdel-Mohsen Hammad M, Osama Abdel-Aziz, Raghda Alsheikh Gh, Ehab Wahby A (2004). Idiopathic giant aneurysm of left atrial appendage. IJTCVS.

[8] Ángeles Tejero-Hernández M, Simona Espejo-Pérez, José Suárez-de-Lezo-Cruz-Conde (2012). Congenital aneurysm of the right atrial appendage in a newborn: a rare anomaly. Scientific letters / Rev Esp Cardiol.

[9] Isil Yildirim (2014). An incidentally diagnosed asymptomatic congenital left atrial appendage aneurysm. Turk Gogus Kalp Dama.

